# Supplementary material for: Predicting visual function by interpreting a neuronal wiring diagram
Source: Nature. 2024 Oct 2;634(8032):113–23. doi: 10.1038/s41586-024-07953-5 (PMC11446822; doi:10.1038/s41586-024-07953-5)
Supplement: Supplementary file 6 — CRF and ERF predictions for individual TmY4 and TmY9 cells. Analogous to Supplementary Data 3, but for TmY target types. Shown are the top four monosynaptic pathways, the strongest pathway passing through each of the top ten intermediary types (ranking from Extended Data Fig. 7), and the trisynaptic pathway Tm1–TmY–Dm3–TmY (see the section entitled Prediction of spatial normalization). [file 41586_2024_7953_MOESM6_ESM.zip › DataS4/TmY9q/720575940625561165.pdf]

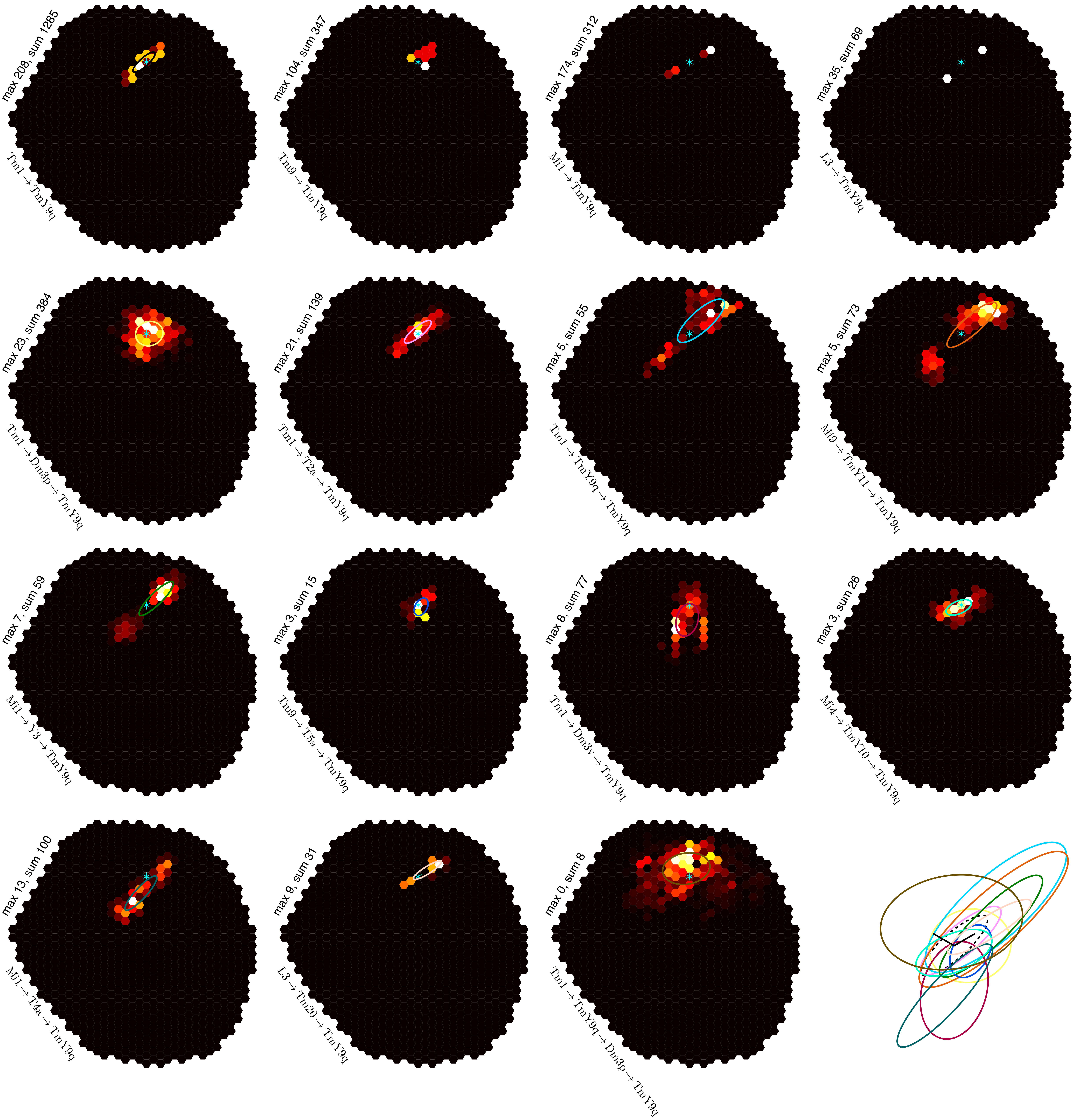
max 208, sum 1285
Tm1 → TmY9q
max 104, sum 347
Tm9 → TmY9q
max 174, sum 312
Mi1 → TmY9q
max 35, sum 69
L3 → TmY9q
max 23, sum 384
Tm1 → Dm3p → TmY9q
max 21, sum 139
Tm1 → T2a → TmY9q
max 5, sum 55
Tm1 → TmY9q → TmY9q
max 5, sum 73
Mi9 → TmY11 → TmY9q
max 7, sum 59
Mi1 → Y3 → TmY9q
max 3, sum 15
Tm9 → T5a → TmY9q
max 8, sum 77
Tm1 → Dm3v → TmY9q
max 3, sum 26
Mi4 → TmY10 → TmY9q
max 13, sum 100
Mi1 → T4a → TmY9q
max 9, sum 31
L3 → Tm20 → TmY9q
max 0, sum 8
Tm1 → TmY9q → Dm3p → TmY9q
